# Different Eye Tracking Patterns in Autism Spectrum Disorder in Toddler and Preschool Children

**DOI:** 10.3389/fpsyt.2022.899521

**Published:** 2022-06-09

**Authors:** Xue-Jun Kong, Zhen Wei, Binbin Sun, Yiheng Tu, Yiting Huang, Ming Cheng, Siyi Yu, Georgia Wilson, Joel Park, Zhe Feng, Mark Vangel, Jian Kong, Guobin Wan

**Affiliations:** ^1^Athinoula A. Martinos Center for Biomedical Imaging, Massachusetts General Hospital, Harvard Medical School, Boston, MA, United States; ^2^Department of Child Psychiatry and Rehabilitation, Shenzhen Maternity and Child Healthcare Hospital, Southern Medical University, Shenzhen, China; ^3^Department of Psychiatry, Massachusetts General Hospital, Harvard Medical School, Boston, MA, United States; ^4^Department of Medicine, Massachusetts General Hospital, Harvard Medical School, Boston, MA, United States

**Keywords:** autism, eye tracking, percentage of fixation time, machine learning, toddler, preschool, support vector machine

## Abstract

**Background:**

Children with autism spectrum disorder (ASD) have been observed to be associated with fixation abnormality as measured eye tracking, but the dynamics behind fixation patterns across age remain unclear.

**Materials and Methods:**

In this study, we investigated gaze patterns between toddlers and preschoolers with and without ASD while they viewed video clips and still images (i.e., mouth-moving face, biological motion, mouthing face vs. moving object, still face picture vs. objects, and moving toys).

**Results:**

We found that the fixation time percentage of children with ASD showed significant decrease compared with that of TD children in almost all areas of interest (AOI) except for moving toy (helicopter). We also observed a diagnostic group (ASD vs. TD) and chronological age (Toddlers vs. preschooler) interaction for the eye AOI during the mouth-moving video clip. Support vector machine analysis showed that the classifier could discriminate ASD from TD in toddlers with an accuracy of 80% and could discriminate ASD from TD in preschoolers with an accuracy of 71%.

**Conclusion:**

Our results suggest that toddlers and preschoolers may be associated with both common and distinct fixation patterns. A combination of eye tracking and machine learning methods has the potential to shed light on the development of new early screening/diagnosis methods for ASD.

## Introduction

Autism spectrum disorder (ASD) is a common neurodevelopmental disorder. Literature suggests that early intervention can significantly impact the prognosis of individuals with ASD and reduce cost of care (1–4). Thus, early detection plays an important role in the treatment of ASD (5, 6).

In recent years, eye tracking (ET), a non-invasive and convenient measurement tool, has drawn the attention of investigators (7–10). The rationale behind applying eye tracking in ASD research is that ASD is associated with different attention patterns from typical development (11–14). Thus, measuring eye movements and gaze patterns using eye tracking technology may help us understand the aberrant behavioral associated with individuals with ASD, and distinguish individuals with ASD from typically developing (TD) individuals.

Faces convey rich personal, emotional and social information starting soon after birth. During even a brief encounter, individuals can automatically attend to and quickly perceive the complex information present in a face, recognizing emotional state and social context, and often remembering the individual face later (15). Brain imaging studies have suggested that eye contact can evoke activity in brain areas in the social brain network, while developmental studies have shown evidence for preferential orienting toward, and processing of faces with direct gaze from early in life (16). Accumulating evidences suggest ASD is associated with an atypical pattern of eye contact behavior (17); thus it is generally agreed that autism involves deficits in face processing. Nevertheless, the precise nature of these deficits and the relationships between anomalous face processing and atypical socio-emotional function in ASD remain unclear (15).

Similarly, biological motion (BM) also conveys information that allows for the identification of affective states and intentions (18, 19). It represents the ability of individuals to detect, label and interpret human movement and to associate certain emotional states with these movements. Thus, biological motion reflects a certain level of social perception (18). Moreover, neurotypically developing individuals have been shown to be able to readily extract socially relevant information from sparse visual displays. Specifically, point-light displays (PLDs), which portray biological motion with points located only on major joints, are readily recognized as depicting differing actions by neurotypically developing individuals, which requires integration across these discrete points of light in order to disambiguate the form (20).

Thus, videos or photographs of human faces and biological motion are the most commonly used in eye tracking paradigms (12, 21–31). With the aid of these paradigms, researchers have identified unique gaze patterns in children with ASD. In a recent meta-analysis, which included 122 independent studies and 1,155 comparisons, Frazier and colleague (21) found a reliable pattern of gaze abnormalities in individuals with ASD, suggesting that individuals with autism have difficulty selecting socially relevant vs. irrelevant information for attention. This problem persists across ages and worsens during perception of human interactions. In addition, the researchers found that although the gaze abnormalities were present across a wide array of stimuli and AOIs, adding perceptual complexity to stimuli or increasing cognitive load during attention-heavy tasks does not increase gaze abnormalities in autism (21).

Nevertheless, few studies have investigated the eye tracking differences across different chronological age groups. Brain imaging studies found that the brain functional and structural alterations associated with ASD may differ across different age groups (5, 6), which indicates that pathophysiology associated with ASD may vary across different ages. In an early study (32), investigators examined temporo-spatial gaze patterns in children (age range 2.8–9) and adults with and without ASD while they viewed video clips. They found that children and adults were separated on the plane, showing a clear effect of age on gaze behaviors. They also found that typically developed infants preferred to watch the mouth rather than the eyes during speech, a preference that reverses later in development. These results highlight the importance of taking the effect of age into account when addressing the gaze behaviors that are characteristic of ASD.

To the best of our knowledge, no study has been carried out to investigate/compare eye tracking patterns between toddlers (ages 1–3) and preschoolers (ages 3–5) with and without ASD. We are particularly interested in these two chronological age groups because despite a clinical diagnosis of ASD, a proportion of the children identified with possible autism before age 3 do not meet criteria for autism at later follow-up (33). Furthermore, although accurate diagnosis of autism spectrum disorder at earlier than 18 months is feasible and overall is more stable than other diagnostic categories (34); earlier diagnosis is associated with many factors such as greater symptom severity, high socioeconomic status, and greater parental concern about initial symptoms (35). Many children are not diagnosed with ASD until age 3 or older despite displaying symptoms much earlier (33, 36–38). Also, 3 years old is cutoff between toddler and preschooler, which represents different developmental milestone.^1^ This calls for intensive investigation of the difference between the toddler and preschooler.

**FIGURE 1:**
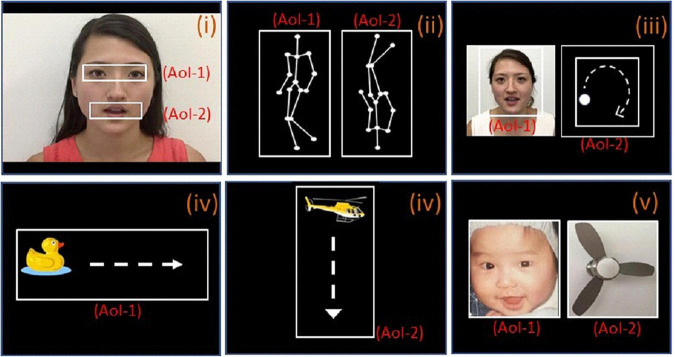
The paradigms used for eye-tracking with area of interest (AOI). **(i)** Female face with moving mouth; AOI-1 and AOI-2 include the eye and mouth regions, respectively. **(ii)** Biological motion; AOI-1 and AOI-2 are point-light display figure of a person walking upright and in the other side, inverted images (upside down), respectively. **(iii)** People and geometry (same size); AOI-1 and AOI-2 are same-sized images of human face with moving mouth and geometry (moving white dot) respectively. **(iv-1)**, a clipart duck moving horizontally from the left side of the screen to the right side, the moving pathway is AOI-1, **(iv-2)**, a clipart helicopter moving vertically from the top of the screen to the bottom, moving pathways is the AOI-2; **(v)**, baby and object (same size); AOI-1 and AOI-2 are same-sized images of baby face and fan respectively.

Elucidating the eye tracking pattern differences between the toddler and preschooler will significantly enhance our understanding of the dynamic characteristics of eye tracking patterns. Thus, the primary aim of this study is to investigate the eye tracking pattern differences between ASD and TD children across toddler and preschool stages using simple paradigms consisting of short videos clips and pictures (Figure 1).

Recently, investigators have tried to use different methods to distinguish ASD from TD in addition to traditional statistical methods, such as the logistic regression model (13). Machine learning techniques, including the support vector machine (SVM), have been applied to identify biomarkers for ASD (39–41). As a subset of artificial intelligence in the field of computer science, machine learning is a procedure that trains the computer algorithm to analyze a set of observed data and statistically learn the latent patterns without being explicitly programmed. One characteristic of SVM is that it does not require any prior assumptions about the underlying relationship between variables (42). In the past, SVM has been used to distinguish between individuals with ASD and TD using multiple measurements such as behavior (43, 44), brain structure (45, 46), activity (47), connectivity (48), and eye tracking (11, 49), and has achieved encouraging results. Thus, this study also aims to explore the eye tracking features that can be used to distinguish ASD from TD across different age groups using SVM.

## Materials and Methods

### Participants

Participants in the ASD group were recruited from the Child Mental Health and Rehabilitation Center of the Shenzhen Maternity & Child Healthcare Hospital in China. Similar to our previous study, inclusion criteria were: (1) aged 1.5–3 or 3–5 years old and (2) Childhood Autism Rating Scale (CARS) score greater than 30 (for those 2 years and older), in line with the ADOS standard. All diagnoses were made by experienced physicians based on the Diagnostic and Statistical Manual of Mental Disorders, Fifth Edition (DSM-5).

TD children were recruited as controls from (1) a kindergarten in the same city or (2) routine annual check-ups in an outpatient ward of the Department of Child Psychiatry and Rehabilitation, Shenzhen Maternity and Child Healthcare Hospital. Specifically, we used the Ages and Stages Questionnaires (Chinese Version) (ASQ-C) (50), Chinese children warning signs checklist (51) and Developmental Screening Parent Questionnaire for Children Aged 0–6 (52) to assess TD children’s communication/language, physical ability, social skills, problem-solving skills, and intelligence. All screenings were performed by a study physician by onsite assessments and communicating with the parents or guardians. Only children that passed the screening, with no history of psychiatric or neurological disorders were included in the study. All participants (ASD and TD) had normal or corrected vision.

A portion of preschool data using paradigm (video clip) data had been used in a previous study to explore whether different parts of body (eye, mouth, nose, hair, and body) could help distinguish ASD from TD (53). However, this study used more comprehensive paradigms and the findings presented in this study have never been published before. The study protocol was approved by the Ethics Committee of the Shenzhen Maternity and Child Healthcare Hospital, Shenzhen, China, and all parents provided and signed informed consent. Informed consent was obtained for publication of identifying information/images in an online open-access publication in the Materials and Methods section.

### Experimental Procedure

Similar to our previous study (53), the SMI RED250 portable eye tracking system was used in data collection. Screen resolution was set to 1,024 × 768 pixels with a sampling frequency of 250 Hz and spatial resolution of 0.03 degrees. Children were seated in front of a 22-inch widescreen LCD monitor in a dark and soundproof room. The center of their vision was aligned with the center of the monitor, with an eye-to-monitor distance of 65 cm. Before the short video presentation, eye position correction was performed by having the subjects fixate on a dynamic pink rabbit (five-point calibration). Those who failed to follow the dynamic pink rabbit fixation did not continue in the experiment. After five-point calibration, silent video clips were presented. The stimulus described below (Figure 1) was presented. Gaze patterns were recorded on a BeGaze data analysis software system. To assess the quality of the data, before the data analysis, the eye tracking instrument Gaze Replay (overall path playback) was used to exclude the subjects whose fixation point deviates from the screen to reduce systematic errors. Only the data of the participants who cooperated, completed the whole experiment and passed the quality check were included in the data analysis.

#### Stimuli

The stimuli consisted of several simple video clips and pictures prepared by co-authors (JP, JK, XJK, copy right obtained to use for study and publication). Please see Figure 1 for details of stimuli and AOIs.

Paradigm i [Video 1 (10 s)]: This video consisted of a woman sitting and mouthing the alphabet without sound. The eyes, which play an important role in social communication and emotional expression, constituted one area of interest (AOI) and the mouth, which represents functional responses related to (early) stages of normative language development, constituted another (11). A similar paradigm (with different background) had been used in a previous study (11).

Paradigm ii [Videos 2a and b (5 s each)]: A point-light display figure of a person walking upright was shown on one side of the screen. On the other side, the same figure was shown rotated 180 degrees, with the person appearing to walk upside down. Each figure was determined as an AOI. A similar paradigm has been used in previous studies (7, 11). Scenario 2b was identical to video 2a, but with the positions of the figures switched (left vs. right side of the screen). The fixation time percentage in 2a and 2b was averaged during data analysis. Preferential attention to biological motion is a fundamental mechanism facilitating adaptive interaction with other living beings. Previous studies have found that children with ASD fail to orient toward point-light displays of biological motion (12, 28).

Paradigm iii [Videos 3a and b (5 s)]: A video of a white dot moving along a circular path (dynamic moving dot) was displayed on one side of the screen, while a video of a woman mouthing the alphabet was shown on the other side (dynamic character). A similar paradigm has been used in a previous study (11). Each video was an AOI with the same size. Video 3b was identical to Scenario 3a, but with the positions of the videos switched. The fixation time percentage in 3a and 3b was averaged during data analysis. This paradigm aims to test if the child favors a moving dot or a woman’s face with a moving mouth to study children’s social interest and social cognition.

Paradigm iv [Videos 4a and b (10 s)]: A clipart duck was shown moving horizontally from the left side of the screen to the right side (5 s, iv-1). This was followed by a clipart helicopter moving vertically from the top of the screen to the bottom (5 s, iv-2). The two moving pathways were the AOIs. The videos aim to test the ability of keeping attention on a moving animal and object.

Paradigm v [Video 5 (5 s)]: An image of an electronic fan was presented next to an image of an infant’s face. The aim of the task is to assess whether the child favors looking at the face or the object (fan) to study children’s social interest and social cognition.

##### Data Analysis

BeGaze software was used for data pre-processing. The software applied filtering, interpolation and smoothing to preprocess the data, including the missing data. The fixation threshold settings were set to duration >100 ms and max displacement <1°of visual angle. Similar to previous studies (11, 54), we used the percentage fixation times allocated to each AOI. The fixation time percentage on each AOI was automatically calculated (time allocated to an AOI/duration of stimulus presentation) by BeGaze and exported to excel file for statistical analysis.

Data analyses were processed with R-studio version 3.5.2. Sociodemographic data between the ASD and TD groups were analyzed using two independent sample *t*-tests and χ2. AOI fixation time percentage analysis was performed with ANCOVA (for each AOI separately). Fix effects include diagnostic group (ASD vs. TD), age group (toddler vs. preschool) and their interaction. Covariate includes age and gender. For each AOI analysis, a test for equality of variance (Levene’s) was applied for Assumption Checks. For AOIs with *p* < 0.05 for the Levene’s test (assumption was not met), weighted least squares (WLS) was applied. *Post-hoc* analysis (Tukey correction) was applied to compare the differences between the preschool ASD vs. the preschool TD, and toddler ASD vs. the toddler TD (except for AOIs of eye and helicopter).

For AOIs of eye and helicopter, the age group by diagnosis group interaction was significant in the ANCOVAs, and therefore the contrasts between ASD and TD and Toddler and Preschool could not be tested using *post-hoc t*-tests. We fit linear regression models for these AOIs, including age-group, diagnosis group and their interaction, and adjusting for age and gender, and calculated estimates and standard errors for the contrasts of interest, using the regression coefficient estimates and estimated variance-covariance matrix of these coefficients from the regression model results. We adjusted for multiple comparisons using the multivariate-t method in the “multcomp” library in “R” (55, 56).

##### Discriminant Analysis

To explore the feasibility of using eye-tracking for the diagnosis of ASD, we applied machine learning with a support vector machine (SVM) to discriminate children with ASD from TD children, using the percentage of fixation time on each AOI as data for this classification, as in our previous publications (48, 53). Since the two cohorts of children (toddler and preschool) exhibited different characteristics in fixation time percentage on different AOIs (see Results for details), we performed the analyses for toddlers and preschool children separately.

Performance of the procedure was assessed by the percentage correctly classified for ASD subjects (sensitivity), and TD subjects (specificity). The accuracy of the procedure was defined to be the percentage of correct classifications combining the two groups. All analyses were based on leave-one-out cross validation by dividing *N* participants (*N* = 95 for toddler; *N* = 74 for preschool) into N-1 training samples and 1 test sample. The same procedure was repeated *N* times to make sure that each participant was used as a test sample once. We then predicted the group assignment for each subject with a separate classifier, determined using only the data from the other subjects, omitting the one predicted. Finally, we determined the performance measures (sensitivity, specificity, accuracy) using these predictions, along with the true group assignments.

## Results

For the toddler cohort, 55 ASD children and 40 age-matched TD children were included in data analysis. For the preschool cohort, 37 ASD children and 41 age-matched TD children were included in data analysis. There were no significant differences in age or gender between ASD and TD in the two age groups (Table 1).

**TABLE 1:** Demographic and clinical traits for all participants (mean ± SD).

Characteristics	Toddler cohort			Preschool cohort		
	ASD (*n* = 55)	TD (*n* = 40)	*P-*value	ASD (*n* = 37)	TD (*n* = 41)	*P*-value
Age (years)	2.4 (0.5)	2.2 (0.6)	0.27	4.6(0.5)	4.8(0.3)	0.11
Gender^†^ (boys/girls)	50/5	33/7	0.22	33/4	33/8	0.29
CARS score	32.3 (1.5)	–	–	33.0 (2.1)	–	–

*^†^The p-value was obtained by χ^2^ test; other p-values were obtained by a two independent samples t-test. CARS, childhood autism rating scale. Of the 55 toddlers, there were eight children who were younger than 2 years old (age ranging from 1.6 to 1.8 years old). The CARS scores were only calculated on children 2 years or older in the Toddler group.*

### Participants’ Fixation Time Percentage at AOIs

ANCOVA showed a significant main effect of diagnostic group (ASD vs. TD) on all AOIs except the moving helicopter, and a significant main effect of age group (toddler vs. preschool) only on the dynamic moving dot. Interestingly, we found significant group and age interaction on fixation time percentage for the eye and moving helicopter AOIs (Table 2).

**TABLE 2:** Fixation time percentage of the areas of interest (AOIs).

AOIs	Toddler cohort		Preschool cohort		Effect of group (ASD vs. TD)	Effect of age (Toddler vs. Preschool)	Effect of interaction
	ASD	TD	ASD	TD			
Mouth^†^	9.1 (13.6)	23.1 (20.3)	11.4 (13.2)	18.0 (16.8)	*F*_(1,167)_ = 15.40, *p* < 0.001	*F*_(1,167)_ = 0.20, *p* = 0.65	*F*_(1,167)_ = 2.21, *p* = 0.14
Eye^#^	15.4 (17.1)	17.6 (20.1)	15.1 (17.5)	29.2 (20.3)	*F*_(1,167)_ = 7.56, *p* = 0.007	*F*_(1,167)_ = 0.19, *p* = 0.67	*F*_(1,167)_ = 3.99, *p* = 0.048
Walking on hands	19.4 (18.1)	35.5 (21.3)	22.1 (19.3)	36.2 (16.9)	*F*_(1,167)_ = 26.41, *p* < 0.001	*F*_(1,167)_ = 2.01, *p* = 0.16	*F*_(1,167)_ = 0.36, *p* = 0.55
Walking upright	20.7 (17.3)	33.3 (20.0)	21.5 (16.5)	28.0 (14.9)	*F*_(1,167)_ = 11.94, *p* < 0.001	*F*_(1,167)_ = 1.14, *p* = 0.29	*F*_(1,167)_ = 1.56, *p* = 0.21
Dynamic character^†^	18.9 (16.1)	32.3 (18.6)	19.8 (18.1)	25.2 (12.1)	*F*_(1,167)_ = 12.75, *p* < 0.001	*F*_(1,167)_ = 0.63, *p* = 0.42	*F*_(1,167)_ = 2.61, *p* = 0.11
Dynamic moving dot	20.1 (16.1)	39.4 (20.4)	25.7 (18.6)	37.5 (17.8)	*F*_(1,167)_ = 32.79, *p* < 0.001	*F*_(1,167)_ = 8.79, *p* = 0.003	*F*_(1,167)_ = 3.62, *p* = 0.06
Helicopter^#^	20.6 (15.4)	16.5 (12.5)	19.8 (14.3)	25.5 (15.0)	*F*_(1,167)_ = 0.21, *p* = 0.644	*F*_(1,167)_ = 0.65, *p* = 0.42	*F*_(1,167)_ = 3.79, *p* = 0.05
Duck^†^	20.0 (17.0)	22.2 (13.9)	19.1 (15.0)	27.6 (13.4)	*F*_(1,167)_ = 5.65, *p* = 0.019	*F*_(1,167)_ = 3.59, *p* = 0.06	*F*_(1,167)_ = 1.03, *p* = 0.31
Baby face	20.0 (18.8)	36.9 (21.6)	21.6 (17.7)	32.1 (16.2)	*F*_(1,167)_ = 22.75, *p* < 0.001	*F*_(1,167)_ = 2.31, *p* = 0.13	*F*_(1,167)_ = 1.72, *p* = 0.19
Electric fan	16.7 (17.0)	19.8 (14.3)	16.9 (16.9)	29.3 (17.0)	*F*_(1,167)_ = 8.80, *p* < = 0.003	*F*_(1,167)_ = 0.67, *p* = 0.41	*F*_(1,167)_ = 2.56, *p* = 0.11

*Data were presented in mean (SD); ^†^Indicates for AOI with p < 0.05 for the Levene’s test (assumption of the equal variance was not met), weighted least squares (WLS) model was applied. ^#^Indicates significant interaction between the diagnosis group and age group. See Results section for results from contrasts of interest using linear regression.*

Because of the significant interactions in the ANCOVAs for eye and helicopter AOIs, we tested the significance of contrast of interest using linear regression, as discussed in the Materials and Methods section. Analysis on the eye AOI showed that *p* = 0.93 for toddler (ASD vs. TD), *p* = 0.006 for preschool (ASD vs. TD), *p* = 0.98 for ASD (toddler vs. preschool), *p* = 0.68 for TD (toddler vs. preschool). For the moving helicopter AOI, *p* = 0.65 for toddler (ASD vs. TD), *p* = 0.31 for preschool (ASD vs. TD), *p* = 0.37 for ASD (toddler vs. preschool), *p* = 1.0 for TD (toddler vs. preschool).

As an exploratory analysis, we all applied *post-hoc* analysis on the ASD and TD differences across different age groups on all AOIs, and calculated the effect size of each comparison, the results are presented in Table 3.

**TABLE 3:** Fixation time percentage differences (AOIs).

AOIs	Toddler (ASD vs. TD)		Preschool (ASD vs. TD)	
	*P*-value	Effect size	*P*-value	Effect size
Mouth	*p* < 0.001	0.81	*p* = 0.25	0.44
Eye^#^	*p* = 0.93	0.12	*p* = 0.006	0.74
Walking upside down	*p* < 0.001	0.81	*p* = 0.01	0.78
Walking upright	*p* = 0.005	0.67	*p* = 0.4	0.41
Dynamic character	*p* < 0.001	0.77	*p* = 0.54	0.35
Dynamic moving dot	*p* < 0.001	1.05	*p* = 0.03	0.65
Helicopter^#^	*p* = 0.65	0.23	*p* = 0.31	0.37
Duck	*p* = 0.86	0.14	*p* = 0.08	0.60
Baby face	*p* = 0.001	0.83	*p* = 0.07	0.62
Electric fan	*p* = 0.80	0.20	*p* = 0.01	0.73

*P-value derived from the post-hoc analysis (Tukey correction) between ASD and TD across different developmental stages. Effect size is measured by Cohen’s d. ^#^Indicates comparisons of interest using linear regression, as described in Materials and Methods section.*

Preferential attention to biological motion is a fundamental mechanism facilitating adaptive interaction with other living beings. Thus, we also investigated biological motion across different age groups. The within-group analysis (paired *t*-test) in four groups (respectively) between the walking upright AOI and walking upside down AOI showed that only preschool TD preferentially attend to biological motion (walking upright) (*p* = 0.01), and there is no significant difference in all other groups (preschool ASD, toddler TD and toddler ASD, *p* = 0.84, 0.59, 0.69, respectively).

To explore the association between gaze fixation time percentage and clinical outcome (CARS), we applied a multiple regression analysis including age and gender as covariates for all ASD children. We found no significant association between fixation time percentage and CARS (*p*-values range from 0.13 to 0.99).

### Discriminant Analysis

Support vector machine analysis showed that the classifier could discriminate ASD from TD in toddlers with an accuracy of 80%, a sensitivity of 80%, and a specificity of 82%; and could discriminate ASD from TD in preschoolers with an accuracy of 71%, a sensitivity of 81%, and a specificity of 61%.

**FIGURE 2:**
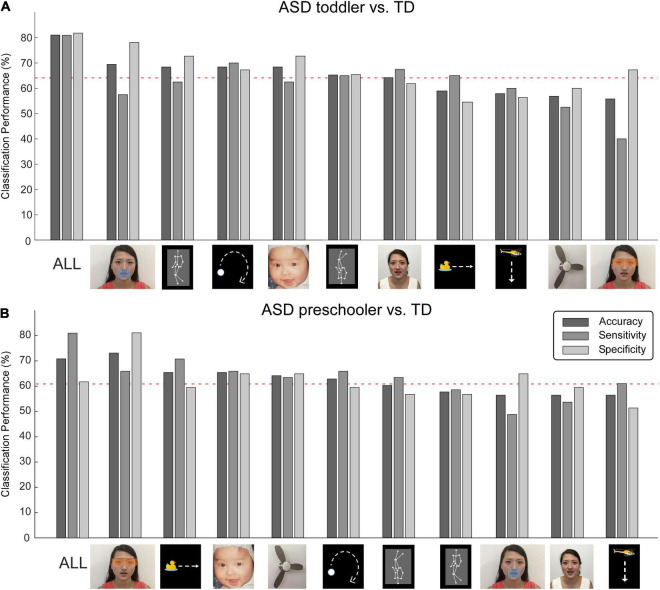
Machine learning accuracies for discriminating autism spectrum disorder (ASD) and typically developing (TD). Classification accuracy, sensitivity, and specificity for all AOIs and 10 single AOIs, for toddlers **(A)** and preschoolers **(B)**, respectively. Red dashed lines represent the 95% confidence interval obtained from non-parametric permutation testing.

Single AOIs, including the moving mouth, walking upright, moving dot, and baby face provided higher accuracies in classification for toddlers. The moving duck, moving helicopter, electric fan picture, and eyes were ranked as the least discriminative features and did not show significant classification accuracies (below the red dashed line in Figure 2A) for ASD toddlers. In contrast, the eyes, moving duck, infant face, and electric fan were the four most important AOIs for discriminating ASD preschoolers from TD preschoolers, and the dynamic character and moving helicopter were ranked as the least discriminative features (Figure 2B) for ASD preschoolers.

## Discussion

In this study, we investigated fixation time patterns between ASD and TD children in toddler and preschool stages. We found that ASD is associated with significant fixation time percentage reduction in most of the AOIs as indicated by the main effect of groups (ASD vs. TD), but only one significant difference between the toddler and preschool groups as indicated by main effect of age group. Interestingly, we also found that toddler and preschool-aged children with ASD are associated with different fixation patterns during the video of the moving mouth (video 1). The fixation time percentage on eye AOI between the ASD and TD children in the toddler is similar, but in the preschool-aged children with ASD, the fixation time percentage on eye ROI is significantly reduced compared to TD (Table 3). The support vector machine analysis showed that the classifier could discriminate ASD from TD with an accuracy of 80%, a sensitivity of 80%, and specificity of 82% in toddlers, and an accuracy of 71%, sensitivity of 81%, and specificity of 61% in preschoolers.

The paradigm 1 is a video clip of a female moving mouth with eye and mouth areas as AOIs. In an earlier study on fifteen male adolescents and young adults with autism and 15 matched control, Klin et al. (7) found that individuals with autism are associated with reduced salience of eyes and increased salience of mouths, bodies, and objects. Fixation times on mouths and objects but not on eyes are strong predictors of degree of social competence. In a following study, Dalton et al. (22) conducted two separate fMRI studies (one with 14 male adolescents with ASD, another with 15 male adolescents with ASD) using eye tracking while measuring functional brain activity during facial discrimination tasks. In both studies, the authors found that activation in the fusiform gyrus and amygdala was strongly and positively correlated with the time spent fixating the eyes in the ASD group, suggesting a heightened emotional response associated with gaze fixation in autism. Following studies provide a more complicated view on eye tracking patterns in ASD. For instance, in a review article on this topic (57), investigators showed that although individuals with ASD often give less preferential attention to social objects and events (faces, people, and social actions), the excessive mouth and diminished eye gaze hypothesis of ASD (58) is not generally supported. Different age groups may be associated with different patterns.

We found significant group and age group interaction on fixation time percentage for the eye AOI. Specifically, we found that for the eye AOI, toddler ASD, and TD is similar, but preschool TD is associated greater fixation time than ASD. For the AOI of the mouth, preschool ASD and TD is similar, but toddler TD is associated with significantly greater fixation time. One potential interpretation is that perhaps toddler TD children tend to take more use of visual mouth information than children with ASD who often have language delays (57). Thus, the toddler TD children pay more attention to the mouth, which in turn reduces attention to the eyes (11). As age increases, the language (sound) itself becomes more meaningful for preschool children. This may explain why preschoolers pay preferential attention to the eyes and have similar attention to the mouth.

In the paradigm ii, we investigated biological motion across different age groups. Preferential attention to biological motion is a fundamental mechanism facilitating adaptive interaction with other living beings. An early study found that 2-year-olds with autism fail to orient themselves toward point-light displays of biological motion (7). In a following study, investigators found that children with autism aged 3–7 years old (28) failed to show preferential attention to biological motion, but age-matched TD do. In our study, we found that only preschool TD preferentially attend to biological motion (walking upright AOI compared to walking upside down AOI). This finding is consistent with the previous study (28), in which investigators found that children with ASD between 3 and 7 years old (28), but not age-matched TD, failed to show preferential attention to biological motion. Our result is only partly consistent with the earlier study on 2-year-old children (7) in which the authors found toddler ASD failed to show preferential attention to biological motion. However, we found that toddler TD also failed to show preferential attention to biological motion, which is not consistent with findings from the previous study (7). It is worth noting that the age range for our study is between 1.5 and 3, while the previous study focused only on 2-year-olds, so we cannot exclude the possibility that the discrepancy is due to age range difference. Further studies are needed to better understand this inconsistency.

In paradigm iii, we compared a white dot moving along a circular path (dynamic moving dot) on one side of the screen and a woman (dynamic character) mouthing the alphabet on the other side. We found a significant main effect on diagnostic group and age (with preschoolers showing greater fixation percentage) on the AOI of the dynamic moving dot in the paradigm. *Post-hoc* analysis indicates there is no significant difference when we analyze the ASD and TD separately in different stages; nevertheless, the effect size is much larger in Toddler group compared to preschool group (1.05 vs. 0.65, Table 3).

Paradigm iv is a video clip of a clipart duck moving horizontally from the left side of the screen to the right side, followed by a clipart helicopter moving vertically from the top of the screen to the bottom. We use this to test attention to moving objects. We found that there is no significant diagnostic group main effect on the helicopter AOI, but a significant main diagnostic group effect on the moving duck AOI. We also found a significant interaction between the group and age on the helicopter AOI, but there is no significant difference detected on *post-hoc* analysis (Table 3). These findings suggest that for some objects such as the moving helicopter, the preferential attention between the ASD and TD is similar in toddler and preschool children with ASD and age-matched TD children.

In paradigm v, we compared an image of an electronic fan and a baby’s face. The results showed that TD is associated with high fixation time percentage as compared to ASD for both AOIs. Further analysis showed that toddler (but not preschool) ASD showed less fixation time percentage to a baby’s face (person related picture). This suggest that only toddler ASD is associated with less preferential attention to social objects and events (faces). We are not sure if this is due to relatively larger sample size in the toddler groups. Further studies are needed to validate this finding.

In this study, we also employed support vector machines (SVM) on fixation time percentage of pre-defined AOIs to discriminate ASD from TD. SVM is a supervised learning model and binary classifier that is used to perform classification analysis. In a study conducted (49) on children from 4 to 11 years old, Liu et al. (49) applied a machine learning method to identify the participants with ASD based on eye tracking data during a facial recognition task, and found they discriminated ASD from TD children with 86.2% specificity and 93.1% sensitivity. Fujioka et al. (11) explored visual fixation time on objects in movies in male adolescents and adults with ASD and found fixation time on objects with large effect sizes for group differences could be used to distinguish ASD from TD participants (sensitivity = 81.0%, specificity = 80.0%). More recently, we (53) conducted an experiment with ASD and TD preschoolers (4–6 year-old) using a moving mouth paradigm (paradigm i) and found that fixation time for the moving mouth and upper body (shoulder) could successfully distinguish ASD from TD (accuracy = 82.8%, sensitivity = 79.3%, specificity = 86.2%).

We found that the classifier could discriminate ASD from TD in toddlers with an accuracy of 80%, a sensitivity of 80%, and a specificity of 82%, and it could discriminate ASD from TD in preschoolers with an accuracy of 71%, a sensitivity of 81%, and a specificity of 61%. Although the paradigm applied in this study produced discriminative accuracy comparable to previous studies, our findings are unique in two respects. First, our study population was between 1 and 3 years old (in the toddler stage), which is much younger than the previous studies mentioned above that applied SVM to distinguish ASD from TD. Toddler years are a crucial period for ASD diagnosis and intervention (33, 36, 37), and the ability to distinguish ASD from TD during toddler years using the machine learning method demonstrates the potential of applying eye tracking for early screening.

Also, a recent meta-analysis (59) on accuracy of Machine Learning Algorithms for the Diagnosis of ASD using Brain Magnetic Resonance Imaging tools showed a sensitivity of 0.83 (95% CI 0.76–0.89), a specificity of 0.84 (95% CI 0.74–0.91) using structural MRI, and a sensitivity of 0.69 (95% CI 0.62–0.75), specificity of 0.66 (95% CI 0.61–0.70) using functional MRI. Our findings based on the eye tracking are comparable with results obtained from sophisticated brain imaging tools. Our results are also comparable with that of studies using traditional statistical methods such as logistical regression analysis. Frazier and colleagues have tried to build an autism symptom index based on eye tracking data collected while the children viewed a 5-min video that included 44 dynamic stimuli from seven distinct paradigms. They found that the autism risk index had high accuracy for ASD diagnosis (area under the curve [AUC] = 0.86), whereas the autism symptom index was significantly associated with ADOS-2 total severity scores (13).

Additionally, we found that the roles of different paradigms in discriminating ASD from TD differ significantly. For instance, the moving duck, helicopter, electronic fan, and eyes (accompanied by moving mouth) cannot be used to distinguish ASD from TD toddlers. Yet, it can be used to distinguish ASD from TD preschoolers. Elucidating the role of different paradigms in ASD classification across different stages may shed light on developing paradigms for earlier ASD detection, particularly for toddlers between 1.5 and 3 years old or even younger.

There are several limitations to this study. First, the ASD diagnoses were made by licensed pediatric psychiatrists based on the DSM-V and CARS (for those 2 years or older), but not confirmed by the ADOS/ADI-R due to limited access to these diagnostic tools in China. Although the TD were screened by licensed pediatrician for psychiatric or neurological disorders and have normal communication/language, physical ability, social skills, problem-solving skills, and intelligence, we did not assess the CARS in TD Children. Second, the sample size was relatively small, and we did not include independent data sets to validate our findings. Thirdly, we did not include developmental assessment or use developmental age in this study. As ASD display significant heterogeneity, predominant ASD studies used chronological age instead of developmental age. Also, in practice, a child transition from Toddler to preschooler is typically based on the chronological age without developmental testing. Thus, we believe that our study of using chronological age is in consistent with previous ASD studies. Future studies may also need to consider adding a developmental test in research on this topic. Forth, we decided not to report positive predictive value (PPV) and negative predictive value (NPV) in this study as it is well-known that PPV and NPV change as prevalence changes. Since the prevalence of autism is very low (1–2%), one can expect a low PPV and high NPV. For example, using the above toddler values of 80% sensitivity and 82% specificity, and assuming 1% prevalence in the population as does the reviewer, the PPV is approximately 4.2%. On the other hand, using these same values, NPV is greater than 99%. Readers should be able to calculate PPV and NPV based on the sensitivity, specificity, and prevalence or *a priori* probability. This decision of not including PPV and NPV is consistent with previous ASD studies on this topic (11, 48, 49). Finally, although we believe that the technology we present is promising and could ultimately lead to a significant improvement in the screening/diagnosis of ASD, this is still an early stage of paradigm development and data analysis for the application of eye tracking in ASD research, the identifying of the most sensitive features to distinguish ASD from TD, and continuous input on this topic is needed for the improvement of clinical implementation of this valuable tool for ASD screening and diagnosis.

In particular, we compared typical cases of ASD with a group of TD with no clear ASD symptoms. Children with a history of psychiatric or neurological disorders were excluded from the TD sample, which may have produced spectrum bias (60). Thus, the ability of the eye tracking to discriminate between the ASD and non-ASD as shown in our study may be overestimated in medical practice. Future studies should include a broad spectrum of the ASD and non-ASD participants to further validate our findings.

In summary, we investigated the fixation time of toddler and preschool children with ASD using different paradigms. Results indicated that toddler and preschool ASD showed both common and unique eye tracking patterns. These findings may shed light on our understanding of the development (age) of ASD and facilitate the development of new screening methods for early ASD detection.

## Data Availability Statement

The raw data supporting the conclusions of this article will be made available by the authors, without undue reservation.

## Ethics Statement

The studies involving human participants were reviewed and approved by Ethics Committee of the Shenzhen Maternity and Child Healthcare Hospital, Shenzhen, China. Written informed consent to participate in this study was provided by the participants’ legal guardian/next of kin. Written informed consent was obtained from the individual(s) for the publication of any potentially identifiable images or data included in this article.

## Author Contributions

X-JK, ZW, GWa, JK, and ZF: experiment design. BS and GWa: data collection. YT, MC, SY, YH, and JK: data analysis. JK, JP, GWi, GWa, MC, and X-JK: manuscript preparation. All authors contributed to the article and approved the submitted version.

## Conflict of Interest

The authors declare that the research was conducted in the absence of any commercial or financial relationships that could be construed as a potential conflict of interest.

## Publisher’s Note

All claims expressed in this article are solely those of the authors and do not necessarily represent those of their affiliated organizations, or those of the publisher, the editors and the reviewers. Any product that may be evaluated in this article, or claim that may be made by its manufacturer, is not guaranteed or endorsed by the publisher.

## Footnotes

1
https://www.cdc.gov/ncbddd/childdevelopment/positiveparenting/
